# Impact of DAXX and ATRX expression on telomere length and prognosis of breast cancer patients

**DOI:** 10.1186/s43046-020-00045-1

**Published:** 2020-08-28

**Authors:** Marwa T. Hussien, Shimaa Shaban, Doaa F. Temerik, Shaaban R. Helal, Eman Mosad, Sahar Elgammal, Abeer Mostafa, Eman Hassan, Abeer Ibrahim

**Affiliations:** 1 Department of Oncologic Pathology, South Egypt Cancer Institute, Assiut University, Assiut, Egypt; 2 Department of Clinical Pathology, South Egypt Cancer Institute, Assiut University, Assiut, Egypt; 3 Department of Clinical Pathology, Faculty of Medicine, Assiut University, Assiut, Egypt; 4 Department of Medical Oncology, South Egypt Cancer Institute, Assiut University, Assiut, Egypt

**Keywords:** Telomere length, DAXX, ATRX, Breast cancer, Prognosis

## Abstract

**Background:**

Telomere stability is one of the hallmarks of cancer that promotes cellular longevity, the accumulation of genetic alterations, and tumorigenesis. The loss of death domain-associated protein (DAXX) and α-thalassemia/mental retardation X-linked protein (ATRX) plays a role in telomere lengthening and stability. This study aims to evaluate the prognostic significance of telomere length (TL) and its association with DAXX and ATRX proteins in breast cancer (BC). Our study used the FISH technique to detect peptide nucleic acid (PNA) in the peripheral blood cells of a cohort of BC patients (*n* = 220) and a control group of apparently healthy individuals (*n* = 100). Expression of DAXX and ATRX proteins was evaluated using immunohistochemistry (IHC) in all BC tissues.

**Results:**

Patients with a shorter TL had worse disease-free survival (DFS) and overall survival (OS). There were significant associations between shorter TL and advanced disease stages, lymph node metastasis, and positive HER2/neu expression. DAXX protein expression was significantly correlated with TL. Lower DAXX expression was significantly with shorter DFS.

**Conclusion:**

Assessing TL can be used as a worthy prognostic indicator in BC patients. Specifically, short TL had a poor impact on the prognosis of BC patients. Low DAXX expression is associated with poor outcomes in BC. Further mechanistic studies are warranted to reveal the underlying mechanisms of these associations.

## Background

Telomeres are dedicated structures located at the chromosomal ends of eukaryotes that act to defend against DNA repair actions and nucleolated degradation [1]. Normally, with subsequent cell division, telomeres shorten, and cells eventually progress to cellular senescence or apoptosis [2]. Telomere length (TL) conservation has been reported to play a key role in continuing everlasting replication and tumorigenesis in cancer cells. It is considered a hallmark of cancer in which transforming cells achieve eternality by upregulating telomerase or by activating the alternative lengthening of telomeres (ALT) pathway. Therefore, cancer cells prevent telomeres from shortening. However, other contradictory studies have shown that short telomeres are associated with a worse prognosis that reflects cumulative cellular aging and genetic instability [3, 4]. Telomere shortening occurs early in the process of carcinogenesis [5–7] and contributes to tumor progression in many cancers including breast cancer (BC) [5, 6]. The common pathway that was reported to linked to ALT in cancer cells occurs through the loss of the remodeling chromatin molecule a-thalassemia/mental retardation X-linked (ATRX) or its binding partner, the histone chaperone death domain-associated protein 6 (DAXX). Subsequently, it induces telomere DNA replication dysfunction, which depends on ATRX/DAXX histone chaperone function [8].

ATRX is a SWItch/Sucrose Non-Fermentable (SWI/ SNF)-like chromatin remodeler that is involved in a scope of nuclear functions including DNA replication, gene expression, and histone variant deposition [9]. The ATRX-DAXX complex is requisite for the integration of the histone variant H3.3 into chromatin [10]. DAXX and H3.3 mutations are also present in tumors with ALT [8]. Either ATRX or DAXX protein inhibition was observed to be strongly correlated with ALT in neuroblastomas, pancreatic neuroendocrine tumors, and sarcomas indicating that the inhibition of these proteins plays vital roles in the initiation of the ALT phenotype [8]. Li et al. [11] found that ATRX and DAXX have no direct function as ALT suppressors. Instead, they revealed that their loss persuades a delayed onset of telomere replication stress that activates the ALT-associated DNA repair pathway while simultaneously compromising mutant cell growth. They proved that this action is distinct from TL and pre-existing endogenous telomerase activity.

DAXX has conflicting roles in BC. Although it plays an important role in tumorigenicity and chemoresistance, it was associated with increased sensitivity to chemotherapy in subgroups of BC patients, particularly in triple-negative (TN) subgroup [12]. DAXX-mediated DNA damage repair defects can enhance the therapeutic effect of the PARP inhibitor in TNBC. Peiffer et al. found that tumors with high levels of DAXX respond better to endocrine therapy [13].

The role of shortening TL and its effect on survival has been confirmed in several studies on BC. We conducted this study to assess the effects of TL, DAXX, and ATRX on BC behavior and prognosis. In this study, IHC was used as a surrogate marker to assess the loss or presence of DAXX and ATRX expression because previous studies proved that their IHC expressions might be rapidly prevailed into routine clinical practice [14].

We also evaluated the TL of peripheral blood leukocytes (PBLs) instead of cancer tissue because the rate of telomere shortening has been reported to be similar in different types of somatic tissues [15, 16]. Notably, Weischer and colleagues [3] examined 47.102 individuals with cancer for 20 years and observed that shorter leukocyte TL was associated with poor survival. Thus, most previous researchers have used PBL PBLs TL instead of tissue sampling for assessing of TL.

## Methods

This prospective study included 220 primarily diagnosed BC patients and 100 controls who were apparently healthy volunteers. All cases were recruited and pathologically diagnosed in our institute, from December 2014 to December 2017. The eligibility criteria included patients who were 20–80 years old, not pregnant, devoid of any other type of cancer, never had any breast surgery including implants or breast reduction, and had been treated surgically with either a mastectomy or breast-conserving surgery.

The control volunteers who were enlisted in this study were females with a similar age as the patients. All volunteers for the control group were female nurses, workers, or doctors who were not first- or second-degree relatives of the patients. All volunteers underwent a mammography accompanied by a breast high-resolution ultrasound (US) prior to obtaining a taking out the blood sample to ensure that they were free from BC.

Whole blood samples were taken from all BC patients and apparently healthy volunteers for the cytogenetic study. The tumors from the BC patients were pathologically staged (pTNM) following the WHO classification of tumors of the breast [17]. The breast specimens were used to evaluate DAXX and ATRX protein expression. Poor prognostic pathologic parameters evaluated included vascular tumor invasion (VI), density of the tumor-infiltrating lymphocytes (TILs), regional lymph node status, and the presence of necrosis. The BC cases ranged from stage I to stage IIIC invasive ductal carcinoma. The cases were molecularly classified according to their HER-2 neu, estrogen receptor (ER), and progesterone receptor (PR) statuses [18].

TILs density can be intratumoral TILs and stromal TILs. The density of TILs in this study was evaluated as the percentage of the tumor stromal area that contains a mononuclear inflammatory infiltrate without direct contact with tumor nests. Density grading varies among studies. In the current study, a 50% cutoff point was used to divide the TILs into focal infiltrate and lymphocyte-predominant breast cancer (LPBC) [19].

A total of 180 patients received adjuvant treatment in the form of four cycles AC (adriamycin cyclophosphamide), and four cycles of paclitaxel, except for four patients who received four cycles of AC only and 30 patients who received neoadjuvant treatment addition to four cycles of AC and four cycles of paclitaxel. We used postoperative tumor tissue samples for the pathological examinations in the patients who received neoadjuvant therapy. All patients who were HER2/neu positive also received trastuzumab at the start of paclitaxel therapy. Ten patients did not receive chemotherapy because they were elderly and presented with early stages of luminal A type BC.

The patient follow-up ended in January 2020. During follow-up, all patients received a physical evaluation every 3 months, an abdominal US and chest X-ray every 6 months, and a routine computerized topography (CT) scan every year or at any time during follow-up if indicated for anything suspicious in the X-ray or US.

### Cytogenetic FISH study

In this study, we used the PNA FISH kit, which contains a telomere PNA probe with a TTAGGG sequence (code K5326, DAKO, Denmark). The heparinized blood samples were cultured in RPMI mixed with glutamine, phytohemagglutinin, 20% fetal bovine serum, and penicillin/ streptomycin. An Axioscope Imager M1 microscope was used for the capture of 20 metaphases of each patient, accompanied by DAPI and Cy3 individual excitation filter, at × 63 magnification, by an attached CCD camera. The ISIS software was used to analyze TL.

Active separation and removing the overlapping of chromosomes was performed, followed by transference to karyotype window. A DAPI automatic banding classifier was used to classify the chromosomes. Telomere measurement area was represented as two horizontal lines overlaid to each chromosome (for p- and q-arms) in the karyogram. The reference signal was measured by applying two horizontal lines on the respective chromosome (chromosome 2). TL was calculated as the ratio between the telomere fluorescence (T) and the centromere fluorescence (C) of chromosome 2 ratio (T/C ratio). C of chromosome 2 has a stable length, so it was used as the internal reference in each metaphase image analyzed [20].

### Immunohistochemistry

Formalin-fixed paraffin-embedded (FFPE) samples were prepared from tumor tissues of the BC patients and underwent an IHC analysis. The stained hematoxylin and eosin slides produced from these blocks were reviewed histologically for confirmation of infiltrating duct carcinoma before IHC marker staining. The FFPE blocks were cut to a thickness of 3 μm and mounted on positively charged slides. The sections were de-paraffinzed and rehydrated. Tris EDTA was used for antigen retrieval in a heated water bath at 90 °C for 45 min. A hydrogen peroxide block was applied. The sections were incubated at room temperature for 10 min. Then, Ultra V Block was applied to the slides for 5 min. Two primary antibodies were applied into two separate sections from tumor tissues: primary goat polyclonal anti-human DAXX antibody (Catalog # DAXX (S-20) sc 7001, Santa Cruz Biotechnology, Inc., USA) and a primary rabbit polyclonal anti-human ATRX antibody (Catalog # ATRX (H-300) sc-15408, Santa Cruz Biotechnology, Inc., USA). Both were used at a dilution of 1/100 (optimum dilution according to the data sheet). The tissue sections were then incubated in a humid chamber for 1 h at room temperature. Immunostaining was performed a universal staining kit “Ultra Vision Detection System Anti-Polyvalent, HRP/DAB (Ready-To-Use)” (Lab Vision Corporation, catalog # TP-015-HD, Fremont, CA 94539-6406, USA) following the manufacturer’s instructions. Biotinylated Goat anti-Polyvalent was applied to the slides at room temperature for 10 min. Streptavidin was applied for 10 min, and diaminobenzidine (DAB) solution was then applied to the slides for 5–10 min. A counterstain for the tissue sections was performed using Mayer’s hematoxylin. Normal gastric and normal prostatic tissues were used as a positive control for DAXX and ATRX, respectively.

### Evaluation of DAXX and ATRX expression

**Fig. 1:**
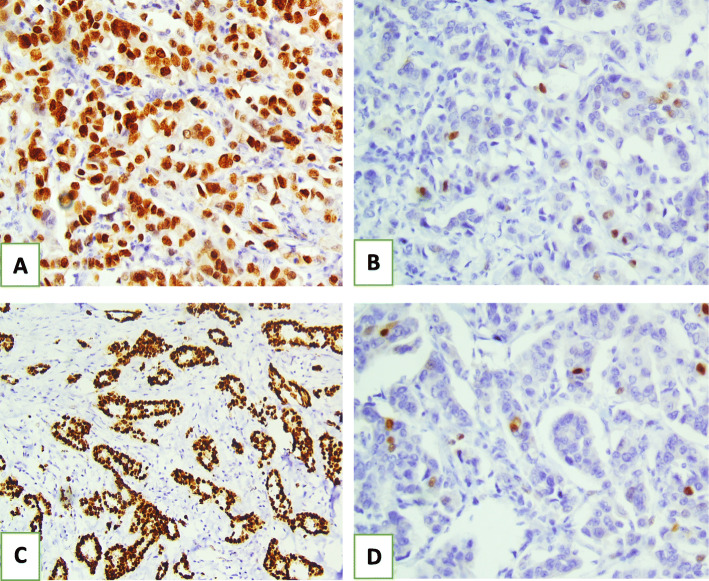
Expression of DAXX and ATRX in tumor cells in invasive duct carcinoma. **a** High expression of DAXX in tumor cells (× 40). **b** Low expression of DAXX in tumor cells (× 40). **c** High expression of ATRX in tumor cells (× 20). **d** Low expression of ATRX in tumor cells (× 40)

An unequivocal moderate and strong nuclear expression with or without cytoplasmic staining of both DAXX and ATRX positivity identified in > 5% of the nuclei of tumor cells is considered positive (high expression) as previously described (Fig. 1) [21]. Positive nuclear staining of DAXX in the gastric gland and positive nuclear staining of ATRX in prostatic ductal cells were used as a positive control. Sections of the tissue-specific positive controls were stained using the same protocol but with omitting the primary antibody, which was used as a negative control.

### Statistical analysis

The statistical analyses were performed by using the statistical package for Social Sciences (SPSS), version 21. The chi-square test was used to detect the associations between each of TL, DAXX protein expression, ATRX protein expression, and various clinicopathological data. An independent *t* test was used to evaluate the associations between TL, DAXX protein expression, ATRX protein expression, and tumor size. A one-way ANOVA test was used to detect the association between TL in the control and early and late tumor stages. Spearman’s correlation coefficient test used to examine the correlations between TL, DAXX protein expression, and ATRX protein expression.

Kaplan-Meier curves and the log-rank survival test were used to analyze overall survival (OS) and the disease-free interval (DFI). *P* < 0.05 was defined as statistically significant.

## Results

This study included 220 BC female patients, all of whom had invasive breast carcinoma of no special type (NST) and 70 (31%) of whom had associated ductal carcinoma in situ (DCIS). There were 8 stage I patients (3.6%), 118 stage II patients (53%), and 94 stage III patients (43.4%). The mean TL was 34.977 ± 10.08. After the median follow-up period of 33 months, 170 patients were still alive (77%). Disease recurrence occurred in 90 cases (42.2%) (Table 1).

**Table 1:** Clinicopathological characteristics of the patients

Variable	Number of cases (%)
**Age**	
< 40 years	86 (39%)
≥ 40 years	134 (61%)
**Tumor size**	
Mean ± SD	4.68 ± 2.15
**Tumor grade**	
**GI**	30 (13.6)
**GII**	130 (59.1)
**GII**	60 (27.3)
**Necrosis**	
Absent	114 (51.8%)
Present	106 (48.2%)
**Vascular invasion**	
Absent	82 (37.3%)
Present	138 (62.7%)
**TILs**	
Focal	64 (29.1%)
LPBC	156 (70.9%)
**Lymph node metastasis**	
N0	90 (40.9%)
N1	40 (18.2%)
N2	36 (16.4%)
N3	54 (24.5%)
**Stages**	
**Stage IB**	8 (3.6%)
**Stage IIA**	84 (38%)
**Stage IIB**	34 (15%)
**Stage IIIA**	22 (10.2%)
**Stage IIIB**	18 (8.3%)
**Stage IIIC**	54 (24.9%)
**Hormonal status**	
ER and PR negative	82 (37.3%)
ER and/or PR positive	138 (62.7%)
**HER2/neu**	
Negative	138 (62.7%)
Positive	82 (37.3%)
**Mol. classification of BC**	
Luminal A	103 (47%)
Luminal B	39 (18%)
HER2/neu +ve	43 (19%)
Triple negative	35 (16%)
**Status**	
Living	170 (77.2%)
Dead	50 (22.8%)
**Recurrence**	
Absent	130 (59.8%)
Present	90 (39.2%)

*ER* estrogen receptor, *HR* hormonal status, *PR* progesterone receptor, *SD* standard deviation

### Telomere length in BC patients and control group

The mean TL was longer in the BC group (mean ± SD = 40.8372 ± 0.81) compared with the control group (mean ± SD = 26.2587 ± 0.23) (*P* = 0.001). Furthermore, the TL was shorter in the late stage patients (mean ± SD = 31.8400 ± 0.73) compared with patients in the early stages (mean ± SD = 31.8400 ± 0.73) (*P* = 0.006) (Table 2).

**Table 2:** Association between telomere length in breast cancer patients in various stages and control groups Telomere

	***n*** of cases (%)	Telomere length (mean ± SD)	***P***	
**Control group**	100	26.2587 ± 0.23		0.001*
**Breast cancer patients**	220	40.8372 ± 0.81	**0.006#**	
Stage I and stage IIA	70 (31.8%)	46.8343 ± 1.18		
Stage IIB–stage IIIC	150 (86.2%)	31.8400 ± 0.73		

One-way ANOVA test was used for this association. # obtained by least significant difference (LSD) of post hoc of ANOVA test

*SD* standard deviation

*Significant

### Correlation between DAXX, ATRX, and telomere length

DAXX protein expression was negatively correlated with TL (*P* = 0.01, *r =* − 0.245) but DAXX protein expression was positively correlated with ATRX expression (*P* = 0.001, *r =* 0.337). There was no significant correlation between TL and ATRX expression (Table 3).

**Table 3:** correlation between telomere length, ATRX and DAXX protein expression

	TL	DAXX	ATRX
**TL**			
**Correlation coefficient**	1.000	**− 0.245**	0.007
**Sig**		0.01*	0.946
***n***	220	220	220
**DAXX**			
**Correlation coefficient**	**− 0.245**	1.000	**0377**
**Sig**	0.01*		0.001*
***n***	220	220	220
**ATRX**			
**Correlation coefficient**	0.007	**0377**	1.000
**Sig**	0.946	0.001*	
***n***	220	220	220

Spearman’s correlation coefficient test was used for this correlation

*TL* telomere length, *DAXX* death domain-associated protein, *ATRX* α-thalassemia/mental retardation X-linked protein, *Sig* significance

*Significant value

### Association of telomere length and clinicopathological parameters

**Fig. 2:**
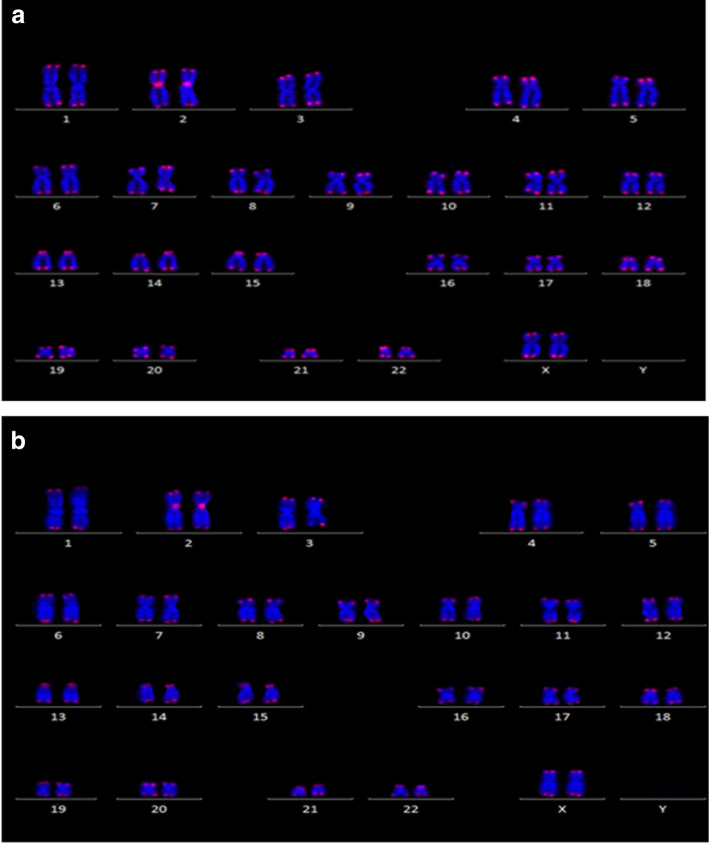
Telomeric signals in relation to tumor stage. **a** Karyogram of early stage tumor shows telomere signals in PBLs. **b** Karyogram of late stage tumor shows telomere signals in PBLs

Telomere lengthening was significantly associated with early-stage tumors (stage I and stage IIA) (Fig. 2a), whereas telomere shortening was associated with late-stage tumors (stage IIB stage IIIC) (Fig. 2b) (*P* = 0.006) (Table 2). The presence of a large tumor size, a high-grade tumor, VI, necrosis, and LPBC were associated with telomere shortening (*P* = 0.003, *P* = 0.011, *P* = 0.004, *P* = 0.001, and *P* = 0.003, respectively) and with lymph node metastasis (LNM) (*P* = 0.001), positive HER2/neu expression (*P* = 0.001), and ER negativity (*P* = 0.03) (Table 4).

**Table 4:** Association of telomere length, DAXX, ATRX expression, and clinicopathological parameters

Variable	Telomere length, ***n***(%)		DAXX, ***n***(%)		ATRX, ***n***(%)	
	Short	Long	Low	High	Low	High
**Age**						
**< 4**0 years	58(66)	30(34)	28(32)	60(69)	50(57)	38(43)
**≥ 4**0 years	98(74)	34(26)	54(41)	78(59)	52(39)	80(61)
***P***	0.346		0.334		0.073	
**Tumor size #**						
*n*	156	64	82	138	102	118
Mean ± SD	4.51 ± 2.24	3.38 ± 1.26	4.41 ± 1.92	4.04 ± 2.15	3.86 ± 1.12	4.46 ± 2.61
***P***	0.003*		0.201		0.056	
**Tumor grade**						
GI	9(30)	21(70)	10(33)	20(67)	12(40)	18(60)
GII	116(89)	14(11)	66(51)	64(49)	71(55)	59(45)
GIII	31(52)	29(48)	6(10)	54(90)	19(32)	41(68)
***P***	0.011*		0.015*		0.032*	
**Necrosis**						
Absent	54(47)	60(53)	26(23)	88(77)	52(46)	62(54)
Present	102(96)	4(4)	56(53)	50(47)	50(47)	56(53)
***P***	0.004*		0.001*		0.887	
**TILs**						
Focal	4(6)	60(94)	16(25)	48(75)	34(53)	30(47)
LPBC	152(97)	4(3)	66(42)	90(58)	68(44)	88(56)
***P***	0.003*		0.88		0.362	
**VI**						
Absent	20(24)	62(76)	16(20)	66(80%)	38(46)	54(54)
Present	136(99)	2(1)	66(48)	72(52)	64(46)	64(54)
***P***	0.001*		0.003*		0.997	
**LN metastasis**						
N0	26(29)	64(29)	22(24)	68(76)	44(49)	46(21)
N1	30(75)	10(25)	18(45)	22(55)	26(65)	14(35)
N2	28(78)	8(22)	20(56)	16(44)	8(22)	28(78)
N3	46(85)	8(15)	22(41)	32(59)	24(44)	30(56)
***P***	0.001*		0.094		0.045*	
**ER**						
**(−ve)**	80(93)	6(7)	20(23)	66(77)	36(42)	50(58)
**(+ve)**	76(57)	58(43)	62(46)	72(54)	66(49)	68(51)
***P***	0.03*		0.015*		0.448	
**HR**						
ER and PR −ve	78(95)	4(5)	16(19)	66(81)	32(39)	50(61)
ER and/PR +ve	78(56)	60(44)	66(48)	72(52)	70(51)	68(49)
***P***	0.002*		0.003*		0.234	
**HER2/neu**						
**(**−**ve)**	74(54)	64(46)	44(32)	94(68)	72(52)	66(48)
**(+ve)**	82(100)	0(0)	38(46)	44(54)	30(37)	52(63)
***P***	0.001*		0.129		0.113	
**Mol. class.**						
Luminal A	43(42)	60(58)	40(39)	63(61)	54(52)	49(48)
Luminal B	39(100)	0(0)	26(67)	13(33)	17(44)	22(56)
HER2/neu +ve	43(100)	0(0)	12(28)	31(72)	13(30)	30(70)
Triple −ve	31(89)	4(11)	4(11)	31(89)	18(51)	17 (49)
***P***	0.002*		0.005*		0.503	

*ER* estrogen receptor, *HR* hormonal status, *PR* progesterone receptor, *DAXX* death domain-associated protein, *ATRX* α-thalassemia/mental retardation X-linked protein. –ve, negative; +ve, positive; *n* number

*Significant

#Independent *t* test used

### Association of DAXX, ATRX, and clinicopathological parameters

DAXX was highly expressed in 138 patients (62.8%), while ATRX was highly expressed in 118 patients (53.6%). DAXX was significantly associated with tumor grade (*P* = 0.015), the presence of necrosis (*P* = 0.001), positive vascular invasion (*P* = 0.003), and positive hormonal receptors (*P* = 0.003). ATRX expression was significantly associated with high-grade tumor and LNM (*P* = 0.032 and *P* = 0.045, respectively). There were no significant associations between DAXX expression and tumor size, TILs, and LNM or between ATRX expression and patient age, tumor size, necrosis, TILs, and hormonal receptor expression. The associations of DAXX, ATRX expression, and clinicopathological parameters are illustrated in Table 4.

### Outcome analysis

**Fig. 3:**
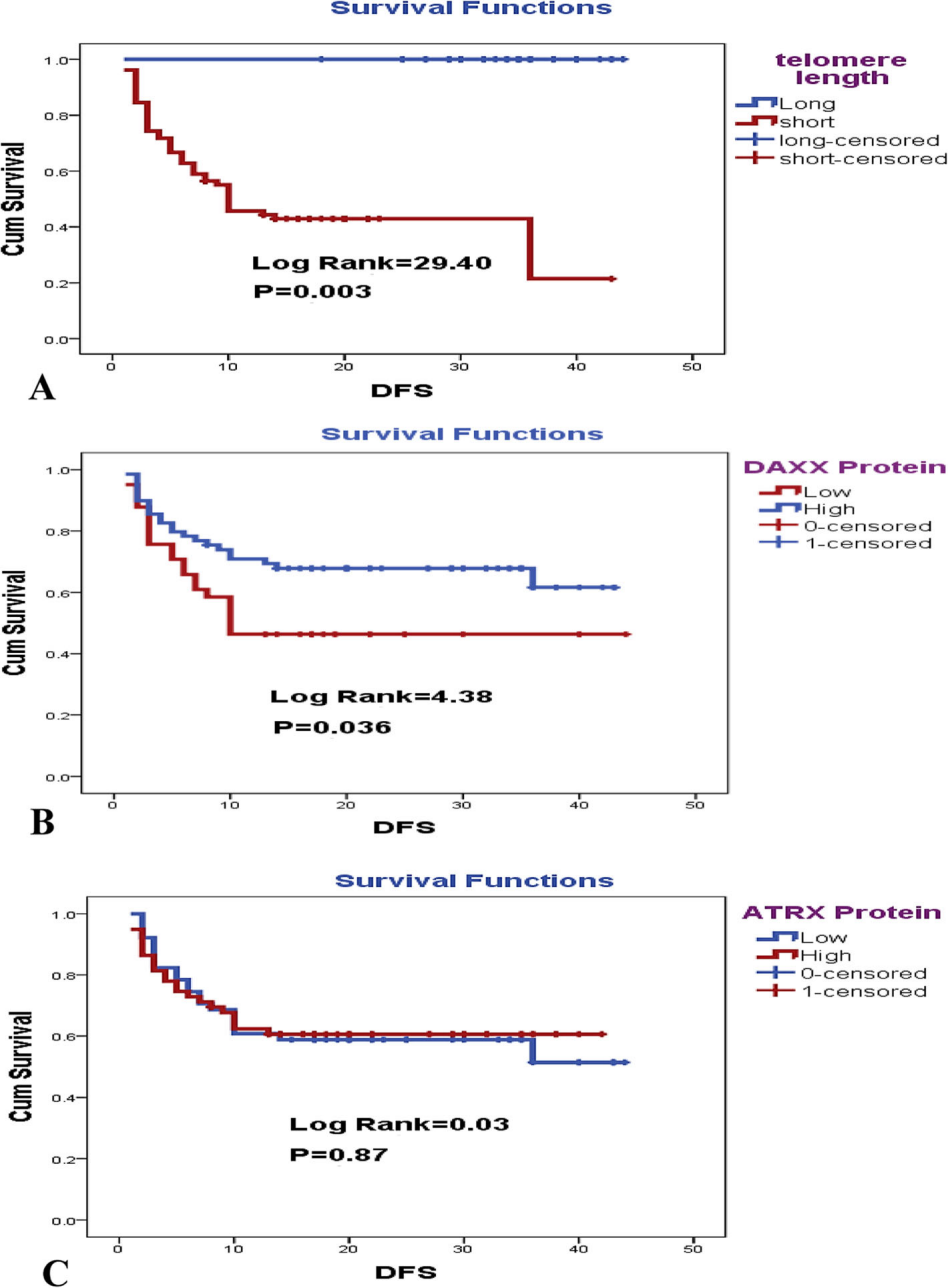
DFS for telomere length, DAXX, and ATRX expression. **a** Telomere shortening is associated with poor DFS. **b** DAXX low expression showed significant bad impact on DFS. **c** ATRX low expression did not show significant impact in DFS

**Fig. 4:**
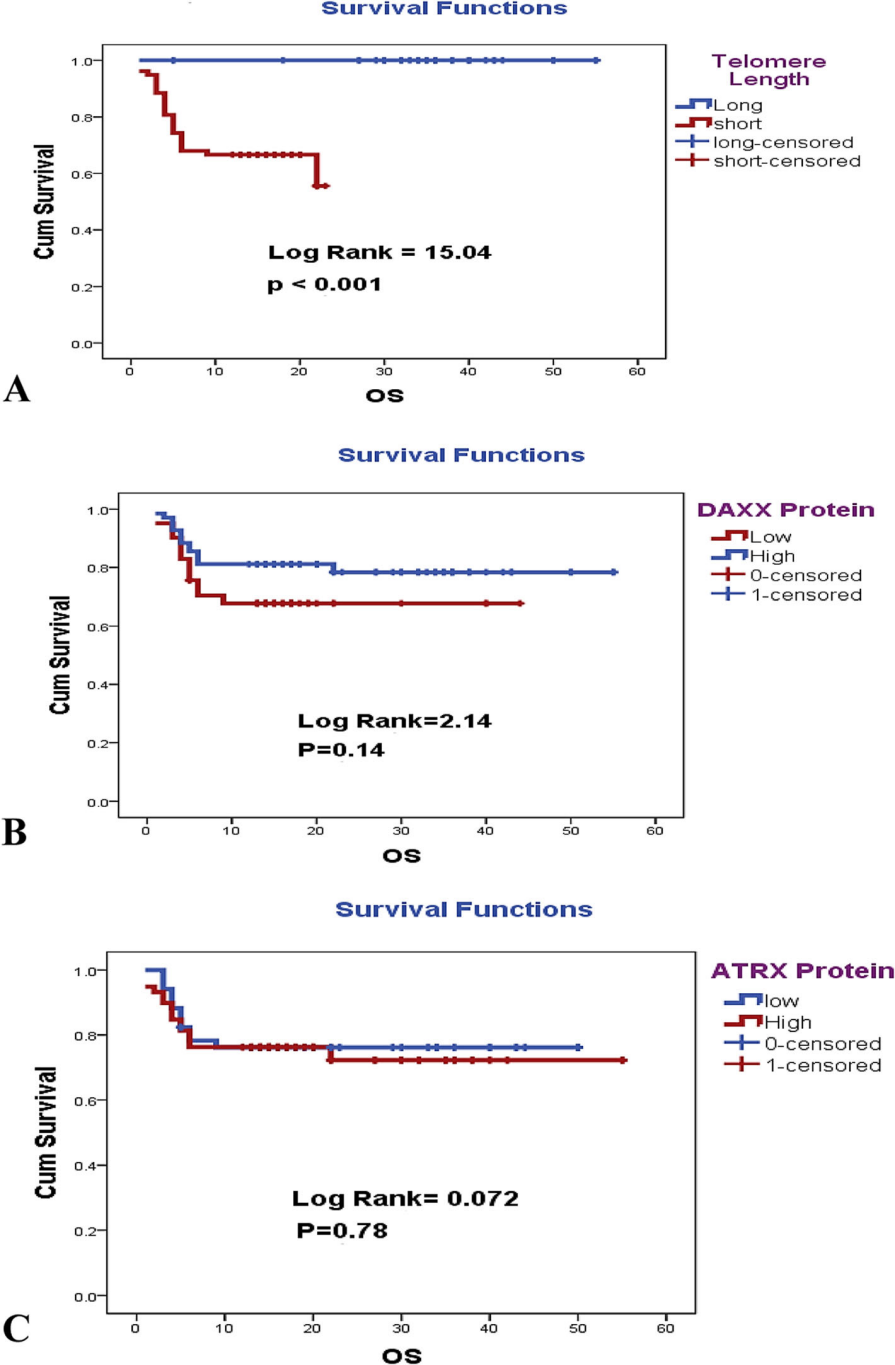
OS for telomere length, DAXX, and ATRX expression. **a** Telomere shortening is associated with poor OS. **b** ATRX and **c** DAXX low expression did not show bad impact on OS

After a median follow-up period of 33 months, 170 patients were still alive (77%). Disease recurrence detected in 90 cases (42.2%).Telomere shortening was significantly associated with poor disease-free survival (DFS) (*P* = 0.003) and OS (*P* = 0.001) (Figs. 3a and 4a, respectively).

Our results could not find any negative impact on survival in patients who were negative for the expressions of both ATRX and DAXX. However, when analyzing ATRX and DAXX independent from one another, low DAXX expression had a significant negative effect on DFS (*P* = 0.036) but not OS (Figs. 4b and 3b, respectively). Conversely, low ATRX expression did not show any statistically significant associations with DFS or OS (Figs. 3c and 4c). Additionally, low DAXX expression was associated with a worse DFS in ER positive BC (*P* = 0.043).

## Discussion

Contradictory roles of TL, DAXX, and ATRX in BC have been reported among different studies [3, 11, 12]. Accordingly, the current study was conducted to measure TL in PBLs and to investigate the expressions of the DAXX and ATRX proteins in the BC tissue.

On the one hand, this study revealed that the TL was significantly longer in the blood leucocytes of patients in the early stages of BC than in the age-adjusted control group. This finding agreed with other large trials [22] but contradicts the results of Barwell et al. [23]. Our explanation of this difference was attributed to the ethnic variations and the methods used for measurement.

On the other hand, we determined that the mean TL was significantly shorter in patients with advanced-stage disease, hormonal negative patients, patients with lymph node metastases, and HER2/neu positive patients. This finding was in agreement with three recent trials that demonstrated that short TL in BC is associated with poor prognostic features [24]. Nevertheless, the observation by Barczak et al. contradicts our results because they reported the increased TL among cases of positive HER2/neu BC. Such a contradiction suggests that there are other mechanisms or associations of TP53 deletion that could be responsible for this extreme difference between studies [25].

Telomere lengthening was significantly associated with positive estrogen expression; this finding agreed with the results of Ennour-Idrissi et al. [26]. The association of longer telomeres with an increased BC risk may be ascribed to an estrogen effect because increased estrogen exposure is a well-known risk factor for BC development. Estrogen affects TL directly through the activation of the human telomerase reverse transcriptase promoter and through by post-transcriptional human telomerase reverse transcriptase regulation [27].

This study identified a negative correlation between DAXX and TL. This was incompatible with other studies suggesting that DAXX inhibition had a negative impact on telomerase-mediated TL control and resulted in telomeres shortening over time [10]. This contradiction caused by the different techniques was used in the measurements DAXX and TL in vitro.

Both DAXX and ATRX expressions were significantly associated with high tumor grades. However, there was no significant association between either DAXX or ATRX and tumor size. This was not in agreement with a study by VandenBussche et al. who reported that the loss of ATRX/DAXX in tumors was associated with a higher tumor grade and large tumor size. This discrepancy referred to the use of a different diagnostic methodology and different tumor origins [28].

We found a significant association between ER-positive patients and low DAXX expression. We did not find any significant association between low DAXX expression and either the expression of progesterone receptors or the co-expression of both estrogen and progesterone receptors. ER/PR-positive E2-mediated ER activation stabilizes the DAXX protein. The stable DAXX protein binds to the regulatory regions of pluripotent and other stem cell genes, possibly recruiting DNMT1 to hypermethylated promoter or gene body regions, resulting in the repression of gene transcription. Thus, DAXX protein stability is potentially responsible for restricting tumor-initiating cell (TIC) survival and frequency. The targeted inhibition of ER has resulted in the rapid depletion of the DAXX protein, the loss of DAXX enrichment at the regulatory regions of stem cell genes, hypomethylation of the SRY-box 2 (SOX2) promoter and partly the NOTCH4 gene, and increased TIC survival. DAXX could be a novel TIC suppressor because the ectopic expression of DAXX reduced pluripotent and stem gene expression, NOTCH signaling, and TIC survival when cells were treated with endocrine therapy [29].

A significant association was found between low DAXX expression and poor DFS. This might be explained by an increase in taxon resistance because almost all the patients in this study received paclitaxel in their adjuvant treatment. Previously DAXX was confirmed as a novel controller of taxanes response in animal models, cell cultures, and human tumor tissues. As previously described, the reduction of Taxol responses was noted with the experimental modification of DAXX levels in both human larynx carcinomas and breast cancer cells [29].

Recently, Giovinazzi S found that tumors with the loss of both RAS-association domain family protein 1 (Rassf1) and DAXX exhibited high mitotic and interphase indices. Thus, the cells were competent for maintaining the mitotic block through raised cyclin B steadiness and proceeding with proliferation after Taxol decay, which occurs quickly in nude mice. Subsequently, mitotic cells from DAXX- and Rassf1A-exhausted tumors can conceivably reenter the G1 phase after drug decay, which suggests a working model as to how cells or tumors without these protein targets can survive chemotherapy treatment and multiply [30].

In summary, TL can be measured in PBLs via a noninvasive technique. Additionally, TL has a valuable role as a prognostic marker. Thus, TL should be assessed TL in all BC patients. Further studies are recommended with a larger number of BC cases to better evaluate the roles of DAXX and ATRX proteins in carcinogenesis, disease progression, and their underlying mechanisms. Furthermore, analyses of the interactions of other molecules with DAXX and ATRX proteins, e.g., the histone variant H3.3 should be evaluated in BC.

## Conclusion

Using the FISH technique to measure TL from PBLs may play a prognostic tool in reflecting the alterations of TL in various breast cancer stages; it can also play valuable role in evaluating survival. DAXX depletion may be important role in both chemotherapy and endocrine therapy resistance. Moreover, a negative correlation was found between TL and DAXX expression, whereas a positive correlation was found between DAXX and ATRX expressions in tumor cells. Therefore, both ATRX and DAXX are included together in a pathway that plays a role in cancer development mechanisms. However, a subsequent study with a larger study population is recommended to confirm these results.

## Data Availability

The datasets used during the current study are available from the corresponding author on reasonable request.

## Abbreviations

DAXX: Death domain-associated protein
ATRX: α-thalassemia/mental retardation X-linked protein
TL: Telomere length
BC: Breast cancer
ALT: Alternative lengthening of telomeres
PNA: Peptide nucleic acid
PBLs: Peripheral blood leucocytes
IHC: Immunohistochemistry
DFS: Disease-free survival
OS: Overall survival
TN: Triple negative
TILs: Tumor-infiltrating lymphocytes
LPBC: Lymphocyte-predominant breast cancer
ER: Estrogen receptor
PR: Progesterone receptor
FFPE: Formalin-fixed paraffin-Embedded
VI: Vascular invasion
LNM: Lymph node metastasis
Rassf1: RAS-association domain family protein
TIC: Tumor-initiating cells
SOX2: SRY-box 2
